# Mitochondrial Metabolism, Redox, and Calcium Homeostasis in Pulmonary Arterial Hypertension

**DOI:** 10.3390/biomedicines10020341

**Published:** 2022-02-01

**Authors:** Shuxin Liang, Manivannan Yegambaram, Ting Wang, Jian Wang, Stephen M. Black, Haiyang Tang

**Affiliations:** 1 State Key Laboratory of Respiratory Disease, National Clinical Research Center for Respiratory Disease, Guangdong Key Laboratory of Vascular Disease, Guangzhou Institute of Respiratory Health, The First Affiliated Hospital of Guangzhou Medical University, Guangzhou 510120, China; lsx2020@gzhmu.edu.cn (S.L.); jiw037@ucsd.edu (J.W.); 2 College of Veterinary Medicine, Northwest A&F University, Yangling 712100, China; 3 Center for Translational Science, 11350 SW Village Pkwy, Port St. Lucie, FL 34987, USA; myegamba@fiu.edu (M.Y.); tinwang@fiu.edu (T.W.); 4 Department of Environmental Health Sciences, Robert Stempel College of Public Health and Social Work, Port St. Lucie, FL 34987, USA; 5 Department of Cellular Biology & Pharmacology, Herbert Wertheim College of Medicine, Florida International University, Port St. Lucie, FL 34987, USA

**Keywords:** metabolism, mitochondria, pulmonary hypertension

## Abstract

Pulmonary arterial hypertension (PAH) is a progressive disease characterized by elevated pulmonary arterial pressure due to increased pulmonary vascular resistance, secondary to sustained pulmonary vasoconstriction and excessive obliterative pulmonary vascular remodeling. Work over the last decade has led to the identification of a critical role for metabolic reprogramming in the PAH pathogenesis. It is becoming clear that in addition to its role in ATP generation, the mitochondrion is an important organelle that regulates complex and integrative metabolic- and signal transduction pathways. This review focuses on mitochondrial metabolism alterations that occur in deranged pulmonary vessels and the right ventricle, including abnormalities in glycolysis and glucose oxidation, fatty acid oxidation, glutaminolysis, redox homeostasis, as well as iron and calcium metabolism. Further understanding of these mitochondrial metabolic mechanisms could provide viable therapeutic approaches for PAH patients.

## 1. Introduction

The 6th World Symposium on Pulmonary Hypertension defines pulmonary hypertension (PH) as mean pulmonary arterial pressure (mPAP) > 20 mmHg at rest in the supine position measured by a right heart catheterization (RHC). Based on similar histopathology, clinical manifestations, hemodynamic characteristics, and therapeutic approaches, PH is classified into five World Symposium Pulmonary Hypertension (WSPH) groups [1]. This review mainly focuses on the first group of PH, pulmonary arterial hypertension (PAH). PAH is progressive and often fatal, in which increased pulmonary vascular resistance (PVR) leads to right ventricular (RV) remodeling and failure, increasing the risk of premature death. Indeed, sustained pulmonary vasoconstriction and excessive obliterative pulmonary vascular remodeling are universal hallmarks of PAH [2].

At a molecular level, dysfunction of various pulmonary vascular cells (PVCs), including endothelial and smooth muscle cells (SMC), is associated with PAH pathobiology [3]. Mechanistically, alterations in cellular metabolism, including aerobic glycolysis, pentose phosphate pathway (PPP), glutaminolysis, and fatty acid (FA) oxidation (FAO), as well as inhibited glucose oxidation in the PVCs, are emerging as triggers of the pulmonary vasculature and RV dysfunction and consequently PAH development. The master transcription factor, the hypoxia-inducible factor (HIF), has been identified as a critical effector in metabolic shift due to its regulation of metabolic enzymes [4]. Our previous work has demonstrated that increased HIF-2α expression in pulmonary vascular endothelial cells plays a pathogenic role in the development of severe PAH [5]. In addition, mitochondrial modulation of reactive oxygen species (ROS), iron metabolism, and calcium homeostasis participate in controlling the vascular remodeling, RV hypertrophy (RVH), and hypoxic pulmonary vasoconstriction (HPV), linked to PAH development [6]. Here, we review the role of metabolic pathways and related mechanisms in PAH and discuss whether these mechanisms may represent potential therapeutic perspectives.

## 2. Mitochondria Morphology and Dysfunction in Pulmonary Hypertension

Mitochondrial morphology is an essential factor influencing mitochondrial ATP synthesis, metabolism signals, redox, and calcium homeostasis. Mitochondria exist as dynamic networks with many forms (small spheres or ovals and short tubules, to elongated tubules and reticular networks in various cell types) depending on whether the mitochondrial network is undergoing fission or fusion, respectively [7]. During the development of diseases, mitochondria display dramatic alterations in mitochondrial morphology, which is associated with the change of fusion or fission pathways. The fusion and fission state of mitochondria is controlled by specific GTPases (OPA1, MFN1, MFN2, and DRP1). It has been reported that mitochondria in SMCs from patients with PAH are fragmented. The molecular mechanism behind the fragmented mitochondria is linked to downregulated fusion proteins (MFN2 and OPA1) and simultaneous upregulation of fission proteins (DRP1) [8]. The molecular mechanism underlying PH lies in the hyperproliferation of cells where the mitochondria actively divide to ensure their equal re-distribution. Further, blocking mitochondrial fission with Trimetazidine prevents the cellular proliferation of pulmonary arterial SMCs and a hypoxia-induced metabolic shift [9]. Understanding the molecular mechanisms underlying mitochondrial morphology alternations that lead to mitochondrial metabolic aberration and dysfunction may provide new approaches for treating pulmonary vascular diseases.

Mitochondrial dysfunction can arise due to aberrant expression of proteins in the electron transport chain, redox-associated enzymes, apoptosis, and mitophagy [10]. As observed in cancer cells, PVCs from patients with PAH exhibit a Warburg effect caused by a shift from mitochondrial oxidative phosphorylation to aerobic glycolysis due to modified mitochondrial bioenergetics. In cells, mitochondria play an essential role in cellular bioenergetics and ROS production [11], while increasing glycolysis leads to altered production of tricarboxylic acid (TCA) cycle intermediates and substrates essential for increased cell growth and proliferation. In PAH, the mitochondria rely on glutamine anaplerosis, the critical mitochondrial metabolic pathway required for active cell growth and proliferation. Further, mitochondria can also derive alternative energy sources from FA synthesis [12]. Researchers are now exploring the relationships between glucose and FA metabolism and are investigating the involvement of multiple metabolic pathways in the development of PAH [13]. With the knowledge that mitochondrial dysfunction leads to decreased glucose oxidation and increased FAO, scientists could reverse the trend, using FAO inhibitors (Trimetazidine and Ranolazine) [14,15]. However, it should be noted that increased FAO is only observed in the later stages of the disease. In the early stages of the disease, decreased FAO is observed, linked to the loss of nitric oxide (NO) signaling and pulmonary endothelial dysfunction [16,17,18,19]. Further, stimulating FAO prevents the development of pulmonary endothelial dysfunction [20,21]. Thus, it will be critical to determine where the PAH development continuum is before FAO modification strategies are employed. More research is required to understand the molecular signals and regulatory checkpoints of mitochondrial dysfunction and the mitochondrial metabolic pathways associated with PH.

## 3. Mitochondrial Metabolic Pathways in Pulmonary Hypertension

**Figure 1:**
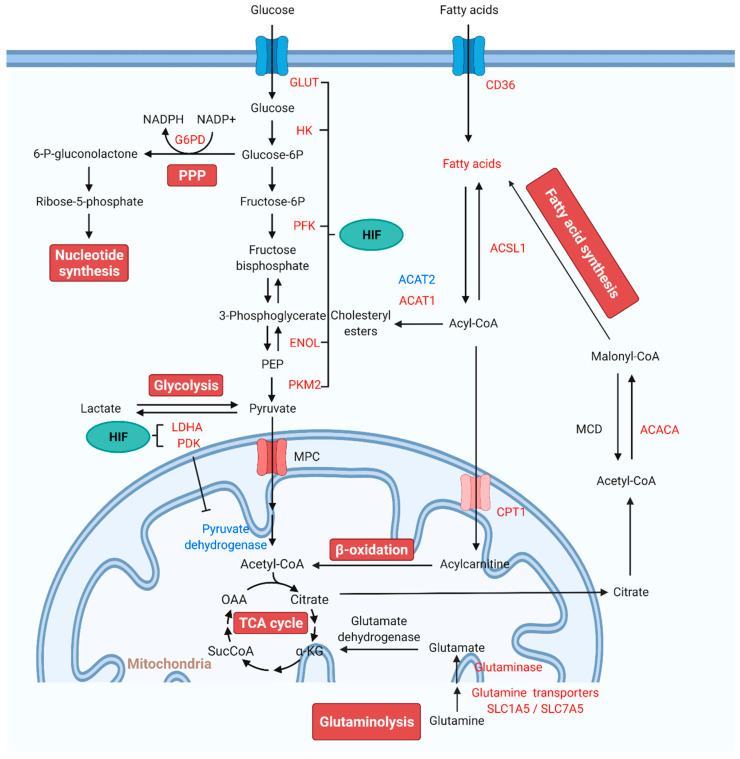
**Alterations of mitochondrial metabolic pathways and therapeutic targets for PAH treatment.** Under normal conditions, glucose is converted to pyruvate via glycolysis. Pyruvate enters the mitochondria, where it is oxidized in the TCA cycle to generate ATP. In PAH, pyruvate is utilized for lactate production. The production of lactate in the presence of oxygen is known as “aerobic glycolysis” or the Warburg effect. In aerobic glycolysis, excess glucose uptake is diverted through PPP. Glutamine is another fuel source, which enters the mitochondria to replenish TCA intermediates and mobilize cellular energy, carbon, and nitrogen. FAs are the main energy source in the healthy adult heart. FA synthesis is started with the formation of malonyl-CoA by carboxylation of acetyl-CoA. Increased FAO inhibits glucose oxidation. HIF activates the transcription of genes encoding metabolic enzymes that mediate the glycolytic pathway. Red indicates increase, and blue indicates reduction. Abbreviations: ACACA, acetyl-CoA carboxylase; ACAT, acetyl-CoA acetyltransferase; Acetyl-CoA, acetyl coenzyme A; ACSL1, fatty acetyl-CoA L1; Acyl-CoA, acyl-coenzyme A; α-KG, α-ketoglutarate; CPT1, carnitine palmitoyltransferase 1; ENOL, enolase; Fructose-6P, fructose 6-phosphate; Glucose-6P, glucose-6-phosphate; G6PD, glucose-6-phosphate dehydrogenase; GLUT, glucose transporter; HIF, hypoxia-inducible factor; HK, hexokinase; LDHA, lactate dehydrogenase A; MCD, malonyl-CoA decarboxylase; MPC, mitochondrial pyruvate carrier; NADPH, reduced nicotinamide adenine dinucleotide phosphate; OAA, oxaloacetate; 6-P-gluconolactone, 6-phosphate-gluconolactone; PAH, pulmonary arterial hypertension; PDK, pyruvate dehydrogenase kinase; PEP, phosphoenolpyruvate; PFKFB, 6-phosphofructo-2-kinase/fructose-2,6-biphosphatase; PKM, pyruvate kinase M; PPP: pentose phosphate pathway; SLC1A5, solute carrier family 1 member 5; SLC7A5, solute carrier family 7 member 5; SucCoA, succinyl-coenzyme A; TCA, tricarboxylic acid.

Recent reports have demonstrated significant alterations in mitochondrial metabolic pathways including glycolysis, glucose oxidation, PPP, glutaminolysis, and FAO. The critical role of mitochondrial metabolic homeostasis in the pathogenesis of PAH is described in detail through these metabolic pathways (Figure 1).

### 3.1. Glycolysis and Glucose Oxidation in Pulmonary Arterial Hypertension

Under normal conditions, glycolysis can convert glucose into pyruvate. Pyruvate is transported to mitochondria via mitochondrial pyruvate carrier (MPC) and then oxidized to acetyl coenzyme A (acetyl-CoA) by the pyruvate dehydrogenase (PDH) complex and finally broken down by the TCA cycle. However, in pathological conditions, such as cancer or PAH, pyruvate is converted to lactate by lactate dehydrogenase (LDH) due to PDH inhibition [22]. Positron emission tomography (PET) imaging using fluorine-18-labeled 2-fluoro-2-deoxyglucose (^18^FDG) shows increased glucose uptake and metabolism in lung tissues and RV of both PAH patients and monocrotaline (MCT)-induced PAH rats, confirming the high glycolytic rate in vivo [23,24,25].

Aerobic glycolysis, a characteristic of non-malignant proliferating cells, has been observed in pulmonary arterial endothelial cells (PAECs) and smooth muscle cells (PASMCs) from idiopathic PAH (IPAH) patients and in rodent PH models [23,26,27]. In IPAH-PAECs, decreased oxygen consumption and increased glycolytic rate underlie the metabolic change from oxidative metabolism to glycolysis in PAH [23]. Less mitochondrial respiration and higher aerobic glycolysis also exists in PASMCs isolated from rats with PH [27]. A similar glycolytic shift has also been observed in RVH [28]. Notably, increased RV ^18^FDG uptake using PET imaging is associated with the severity of disease as shown by higher RV pressure overload and pulmonary artery (PA) pressures, lower cardiac index, and reduced RV systolic function [13,29]. This relationship between ^18^FDG uptake and clinical manifestations suggests that metabolic imaging may play a specific role in PAH clinical diagnosis, either as a prognostic marker or to monitor disease progression and treatment effects [30].

PH has striking similarities with cancer. In the endothelial cells (ECs) and SMCs of the pulmonary vascular wall, PH also elicits cellular glycolytic reprogramming, hyperproliferation, and anti-apoptotic phenotypes [23,31,32]. All the above characteristics are similar to those observed in cancer and precisely highlight the Warburg effect, where glycolytic reprogramming provides the necessary energy for the growth and production of new cells [33,34]. Furthermore, the glycolytic intermediates contribute to the production of phospholipids, nucleotides, and amino acids required to sustain cellular replication. This glycolytic reprogramming modifies the vascular cells in the PAs to proliferate excessively, occluding the arteries and restricting blood flow [35,36]. Establishing a link between cancer and PAH has revealed a common reliance upon mitochondrial dysregulation [37,38,39]. Metabolic and mitochondrial dysregulation are key contributors to the development of PAH as mitochondrial hyperpolarization and the glycolytic shift lead to the upregulation of proteins involved in cellular proliferation and downregulation of apoptotic factors. Similar to cancer, the crucial role of mitochondrial function in the cells of the pulmonary vasculature is the metabolic shift to favor glycolysis to produce ATP and lactic acid fermentation.

A global cellular metabolomics overview suggests that a set of altered mitochondrial metabolites contributes to the pathogenesis of PH [40,41]. Studies have revealed that cells of the pulmonary vasculature have a strong dependence on glutamine, which leads to excessive proliferation [42]. In cells, glutamine is the critical amino acid in several biochemical reactions, and is required for glutamate synthesis by glutaminolysis. Further, glutamate is a substrate for glutathione synthesis. Moreover, it is also converted to α-ketoglutarate, which enters the TCA cycle. Overall, Warburg metabolism modifies the cells to depend on glycolysis to produce ATP and cellular building blocks and further inhibits mitochondria-mediated apoptosis, allowing vascular cells to proliferate.

The pathologic accumulation of hypoxia-inducible factor 1α (HIF-1α), an essential transcriptional regulator of the hypoxic response, plays a critical role in regulating the glycolytic shift from mitochondrial oxidation toward aerobic glycolysis in PAH. Researchers have shown that cultured ECs obtained from patients with idiopathic pulmonary arterial hypertension (IPAH-ECs) have a greater HIF-1α expression [43]. Activation of HIF-1α upregulates the transcription of pyruvate dehydrogenase kinase (PDK, a key enzyme of mitochondrial glucose oxidation), which can inhibit the pyruvate dehydrogenase complex (PDH, a regulator of pyruvate uptake into the TCA cycle), suppressing mitochondrial oxidative phosphorylation and increasing aerobic glycolysis [44]. In PASMCs, RV fibroblasts, and RV cardiomyocytes, activation of PDK promotes the metabolic shift, which contributes to disease pathology [45,46,47,48]. Therefore, PDK has been regarded as a promising target for PAH treatment. Administration of a PDK inhibitor dichloroacetate (DCA) in IPAH patients leads to a reduction in mPAP, PVR, and improvement in RV function [49]. Several studies in PAH animal models have also demonstrated that administration of DCA blocks HIF-1α activation and promotes mitochondrial oxidative phosphorylation, thereby preventing and reversing PAH [48,50,51,52]. In addition to the regulation of glycolytic enzymes in response to hypoxia, HIF-1α can upregulate glucose transporter 1 (GLUT1) and hexokinase (HK) to both increase glucose uptake and retain it within the cell, further supporting a glycolytic shift [30,46,53,54]. HIF-1α also activates the transcription of genes encoding pyruvate kinase M 2 (PKM2), lactate dehydrogenase A (LDHA), enolase (ENOL), and other metabolic enzymes that mediate the glycolytic pathway [55,56]. Another central regulator of glycolysis, the mammalian target of rapamycin (mTOR), is also activated, further shifting cellular metabolism to glycolysis [57]. A differential role of mTOR complex 1 (mTORC1) and complex 2 (mTORC2), two functionally distinct mTOR complexes in PAH, has been identified. The disruption of mTORC1 in SMCs ameliorates the development of experimental PAH; however, disruption of mTORC2 leads to spontaneous PAH [58], which indicates that the mTOR signaling pathway is a critical modulator in PAH development. It should be noted that HIF1 and HIF2 can activate overlapping and different genes in different cell types [59], and HIF-2α mediated gene sets differentiate PAH [60]. Importantly, HIF-induced altered glucose metabolism is essential for the production of ATP and limiting mitochondrial ROS (mt-ROS) production. The Warburg effect has been reported in many PAH and RV failure cases and hypoxia-induced PH, leading to the conclusion that pharmacological modification of Warburg effect-mediated pathological phenotype may be a possible strategy to inhibit PAH.

The rate-limiting enzymes in the glycolytic pathway also participate in the glucose metabolic shift in PAH. The 6-phosphofructo-2-kinase/fructose-2,6-bisphosphatase 3 (PFKFB3) catalyzes the conversion of fructose-6-phosphate (F-6-P) to fructose-2,6-bisphosphate (F-2,6-P2), one of three rate-limiting enzymes in glycolysis. PFKFB3 expression is increased in PASMCs and PAECs in both PAH rodents and IPAH patients. Knockdown of PFKFB3 decreases glycolysis, glycolytic capacity, and glycolytic reserve in PVCsfrom IPAH patients. Endothelial PFKFB3 or SMC-specific PFKFB3 deficiency attenuates hypoxia-induced PAH and vascular remodeling. Likewise, PFKFB3 inhibitor 3PO ameliorates MCT- or Sugen 5416/hypoxia (SuHx)-induced pathological changes in hemodynamics, pulmonary vessels, and RV [61,62].

### 3.2. The Pentose Phosphate Pathway in Pulmonary Arterial Hypertension

Similar to cancer cells, there are ways to increase glucose uptake in PAH through other biosynthetic pathways such as PPP [63]. PPP generates reduced nicotinamide adenine dinucleotide phosphate (NADPH) and ribose-5-phosphate to preserve nucleotide synthesis and redox homeostasis. Increases in PPP flux in PAH patients and multiple animal models have been identified and shown to be associated with the metabolic changes that precede the development of PAH [64,65].

Glucose-6-phosphate dehydrogenase (G6PD) is the first rate-limiting enzyme of the PPP. The increased activity and expression of G6PD have been shown in hypoxic PAs [66], lung tissues of MCT-treated rats [67], endothelin-1 (ET-1)-treated PASMCs [68,69], and hypoxic CD133(+) progenitor cells that maintain cells in high proliferative state [70]. These results indicate that G6PD deficiency may protect against the development of PAH. Indeed, inhibition and knockdown of G6PD in chronic hypoxia-induced PAH and SuHx-PH rat models reverses the metabolic changes, epigenetic modification (DNA methylation), maladaptive expression of genes that contribute to PA remodeling, the formation of occlusive lesions, and RV pressure overload [70,71,72,73]. Protein kinase G1 (PKG1) signaling has a vital role in this process, as G6PD inhibition evokes PKG1α-dependent signaling, thereby mediating expression of contractile proteins, reducing Ang II-induced contraction and proinflammatory factor in PA response to hypoxia [66,74,75]. Inhibition or silencing of G6PD activity induces relaxation of pulmonary and coronary arteries. It attenuates acute HPV via regulating Ca^2+^ signaling [66] and opening of voltage-gated K^+^ (K_v_) channels [76], indicating a positive linear relationship between G6PD activity and HPV [74]. In addition, a deficiency in G6PD activity in the African, Middle East, and Asian populations has been associated with increased susceptibility to hemolysis [77]. Consistently, PAH patients have significantly decreased G6PD expression and activity, indicating that it may be involved in PAH pathobiology due to increased hemolysis [78].

NADPH is a cofactor for the critical antioxidant enzymes glutathione reductase and thioredoxin reductase, which catalyze the conversion of oxidized glutathione (GSH) and thioredoxins (TRX) to their reduced forms, respectively. NADPH is also a critical cofactor for nitric oxide synthase (NOS), maintaining NO synthesis, and, for NADPH oxidase (NOX) enzymes, generating ROS. The activity of NADPH, NADPH/NADP^+^ ratio, and NADPH levels are increased in pulmonary microvascular endothelial cells (PMVEC), containing a bone morphogenetic protein receptor type 2 (BMPR2) mutation [65]. A similar phenotype is observed in PASMC [66] and hypoxic PA and lungs [79]. Overproduction of NADPH can cause damaging “reductive stress” in the cardiovascular system [80]. Increases in NADPH levels inhibit redox activation of PKG in the PA in response to hypoxia, which supports phenotypic modulation of PASMCs from a contractile to proliferative phenotype [66]. Meanwhile, generation or maintenance of cytosolic NADPH by G6PD regulates PA vasomotor tone and HPV via inhibition of K_v_ channels and maintenance of the NO-soluble guanylate cyclase (sGC) pathway [79]. In addition, under pathological conditions, including PAH, the activity of NOXs can be enhanced via utilizing NADPH as a substrate, thereby generating superoxide and hydrogen peroxide (H_2_O_2_) [81]. These products participate in chronic obstructive pulmonary disease (COPD)-related and MCT- and hypoxia-induced vascular remodeling [82,83,84] and vasoconstrictor responsiveness [85].

### 3.3. Glutaminolysis in Pulmonary Arterial Hypertension

Glutaminolysis involves deamination of glutamine to glutamate via glutaminase (GLS) and subsequent conversion of glutamate to α-ketoglutarate (α-KG) by glutamate dehydrogenase. Glutaminolysis contributes to the anaplerosis reaction, whereby the carbon intermediates of the TCA cycle are replenished, and allow cellular energy, and carbon and nitrogen mobilization, especially in the hyperproliferative cells [86].

Increased glutaminolysis in PAH has been found to produce substrate to meet the high energy requirement in hyperproliferative and antiapoptotic PVCs. Specifically, in PVCs and lung tissue of various PAH types, vascular extracellular matrix (ECM) stiffening, an early and potent pathogenic trigger, leads to the yes-associated protein 1 (YAP) and transcriptional coactivator with PDZ binding motif 1 (TAZ) mechanoactivation. Their activation upregulates metabolic enzymes, including GLS1, resulting in glutaminolysis and glycolysis. Anaplerotic production of glutamate and aspartate via GLS1 sustains vascular cell proliferation and migration in a stiff ECM. GLS1-dependent inhibition of glutaminolysis with pharmacologic inhibitors decreased PVC proliferation in vivo and improved manifestations of PAH [87]. Augmented glutaminolysis in PAH promotes lung fibrosis. Glutaminolysis-induced collagen translation and stability by α-KG-mediated mTORC1 activation and collagen proline hydroxylation trigger vascular fibrosis and stiffening, respectively [88]. Pulmonary vascular remodeling is also driven by increased glutaminolysis and glutamate production. An animal model study showed that deficiency or pharmacological blockade of N-methyl-d-aspartate receptor (NMDAR) improves hemodynamics, vascular and cardiac remodeling, and associated PAH. Type A-selective endothelin receptor (ET_A_R) activation or membrane depolarization through K_v_ channel inhibition enhances calcium-dependent glutamate release from PASMCs [89]. In addition, BMPR2 mutations in PAH patients exhibit a shift to glutamine metabolism characterized by large amounts of glutamine uptake. Thus, increased glutamine metabolism required by the endothelium can maintain the energy needs of hyperproliferative cells in PAH [90].

Increased glutaminolysis has been observed in MCT-induced RVH and associated with the maladaptive RVH. The glutaminolysis inhibitor 6-diazo-5-oxo-l-nor-leucine (DON) reduced RVH, and improved cardiac function and treadmill distance in MCT-induced RVH. Moreover, elevated glutamine transporters (SLC1A5 and SLC7A5) suggest an increased glutamine uptake in MCT-induced RVH [91]. Thus, targeting abnormal glutamine metabolism represents a promising therapeutic intervention in treating various forms of PAH.

### 3.4. Altered Fatty Acid Oxidation in Pulmonary Arterial Hypertension

FAO is a primary cellular energy source in normal adult hearts, supplying approximately 60–90% of ATP for contractile function. The remainder is provided by carbohydrates (glucose and lactate) and ketone bodies oxidation [92]. Various cardiac pathological conditions can disrupt FA metabolism, which contribute to a decrease in cardiac efficiency, contractile dysfunction, and hypertrophy [93,94,95,96]. Increased circulating free FAs and RV lipid deposition in the form of long-chain FAs, triglycerides, diacylglycerols, and ceramides associated with lipotoxic cardiac steatosis as well as increased FAO have been observed in PAH patients [13,93,97,98]. Impaired RVH and triglyceride and ceramide deposition are found in *Bmpr2* mutant mice and PA banded rats [93,99]. Increased expression and redistribution of CD36, a FA transporter responsible for FAs uptake into sarcolemma from the intracellular compartment, has been identified in the RVs and cardiomyocytes of the *Bmpr2* mutant mouse [100]. FAO and glucose oxidation share a reciprocal relationship called the Randle cycle, in which the activation of FAO results in the inhibition of glucose oxidation and vice versa. Specifically, when glucose oxidation is suppressed, energy is still produced in the mitochondria via converting carbohydrates (pyruvate) to FAs. Later, FA is transported into mitochondria where it undergoes β-oxidation to produce acetyl-CoA, which enters the TCA cycle. Therefore, Trimetazidine or Ranolazine, partial inhibitors of FAO, restore glucose oxidation, reduce RV hypertrophy, and improve RV function in PA banding rat [99].

Metabolomic results have identified abnormal FA metabolism and accumulation in the pulmonary vasculature in tissues of PAH patients. In particular, dicarboxylic FAs such as tetradecanedioate, hexadecanedioate, and octadecanedioate are found. Moreover, expression of aldehyde dehydrogenase (ALDH), a key enzyme of ω-oxidation, is higher in lung tissues, SMCs, and ECs from patients with PAH, suggesting that ω-oxidation is the main FA oxidation metabolic pathway when β-oxidation is no longer sufficient to supply energy for the pulmonary vascular remodeling in PAH. Several genes encoding enzymes that are involved in FAO, such as fatty acetyl CoA L1 (ACSL1), acyl-CoA dehydrogenase (ACADM), acetyl-CoA acetyltransferase 1 (ACAT1), and acetyl-CoA carboxylase (ACACA), are increased, and ACAT2 expression is decreased in the lungs of PAH patients [101,102]. Importantly, inhibition of FAO may have a beneficial effect in preventing PAH pathogenesis. Lack of malonyl-CoA decarboxylase (MCD) inhibits FAO and converts the metabolic balance to glucose oxidation in vascular media. In animal models, MCD deficiency does not lead to HPV and prevents the development of chronic hypoxia-induced PAH. Trimetazidine and DCA that mimic MCD deletion reduce mPAP, RV hypertrophy, and vascular remodeling, and reverse PAH induced by hypoxia or MCT [103]. Additionally, carnitine palmitoyltransferase 1 (CPT1), a rate-limiting enzyme responsible for transporting acylcarnitine into the mitochondria in the FAO metabolism, is also increased in rat lungs and PAs of the MCT-induced PH model. Enhanced CPT1-driven FAO increased ATP production in lung tissues and promoted PASMC proliferation. At the same time, inhibition of CPT1 activity decreased the level of free FAs entering the mitochondria for FAO, thereby reducing the abnormal accumulation of PASMC [104]. Thus, FA metabolism represents a therapeutic target to treat PAH.

## 4. Redox Homeostasis in Pulmonary Hypertension

ROS, including hydroxyl radicals, superoxide, and H_2_O_2_, exert a crucial role in preserving redox homeostasis [105]. Several enzyme systems regulate ROS formation in the vasculature, including NOXs [106]. Initially, NADPH represents a pivotal reducing equivalent, which is utilized as a substrate to promote NOX activity, thus contributing to pro-oxidant reactions. In addition to the role of the NOX4 subtype in producing H_2_O_2_, vascular NOXs release large amounts of superoxide by catalyzing the transfer of single electrons from NADPH to molecular oxygen [107]. NOX homologs are differentially expressed in vascular ECs, SMCs, fibroblasts, or perivascular adipocytes [108]. NOXs-derived ROS has a regulatory role in the pathophysiological function of pulmonary vessels. NOX1 and NOX2 have been reported to contribute to the progression of endothelial dysfunction, inflammation, and hypertension [109,110]. Abnormal expression and activity of NOX4 can cause oxidative stress, senescence, aortic stiffening, and endothelial dysfunction [111,112]. Calcium-dependent NOX5 has been implicated in angiogenesis, vascular remodeling, and calcification [113,114].

Under conditions where glycolysis and PPP are enhanced and mitochondrial respiration is suppressed, NOX4 expression and activity are significantly elevated [45,82,84]. NOX4 inhibition reduces adventitial ROS generation, vascular remodeling, and PA stiffness, and ameliorates RV hypertrophy, preventing MCT-induced PAH development [82]. NOXs are also oxygen sensors in the lung. NOX4 levels are increased in mouse pulmonary vessels and PASMCs exposed to hypoxia [115]. HIF-1α transcriptionally regulates NOX4 expression. HIF-1α-dependent NOX4 induction contributes to ROS generation and PASMC proliferation and migration after hypoxia [116]. A lack of the gp91^phox^ NOX subunit in mice reduces chronic intermittent hypoxia-induced increases in NOX4 and attenuates PAH development [117]. However, Veith et al. reported that the NOX4 knockout does not affect the response to acute HPV or chronic hypoxia-induced PAH [118]. Increased expression of NOX2 in response to simultaneous treatment with morphine and HIV-Tat has been observed in ECs [119]. However, the role of NOX2 in PAH remains unclear, and it is speculated that it may exert effects on inflammation [120].

**Figure 2:**
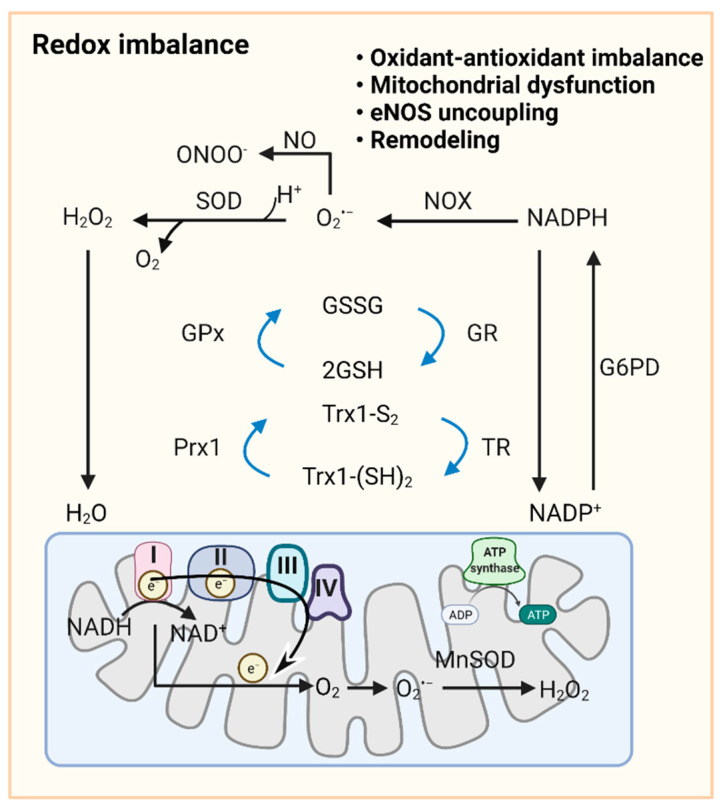
**Redox signaling in pulmonary vasculature.** GSH and Trx1-(SH)_2_ are oxidized to GSSG and Trx-S2, recycled in the redox cycle via the NADPH-dependent enzyme GR or TR, while NADP^+^ can be reduced to NADPH by G6PD in the cytoplasm. Once O_2_^•−^ is formed by cytoplasmic NOXs and mitochondrial ETC, cytoplasmic SOD and mitochondrial MnSOD catalyze its dismutation into H_2_O_2_. GPx uses GSH to further reduce H_2_O_2_ to H_2_O. NO reacts with O_2_^•−^ to form ONOO^−^. In mitochondria, ~0.1~0.2% of the total oxygen accepts electrons from ETC to form O_2_^•−^. H_2_O_2_ is formed by the conversion of O_2_^•−^ catalyzed by MnSOD or spontaneous dismutation. The production of excessive superoxide free radicals can lead to redox imbalance, mitochondrial damage, uncoupling of eNOS, and ultimately to impaired PAdiastolic function and remodeling. Abbreviations: ADP, adenosine diphosphate; ATP, adenosine triphosphate; eNOS, endothelial NO synthase; ETC, electron transport chain; G6PD, glucose-6-phosphate dehydrogenase; GPx, glutathione peroxidase; GR, glutathione reductase; GSH, reduced glutathione; GSSG, oxidized glutathione; H_2_O_2_, hydrogen peroxide; MnSOD, manganese superoxide dismutase; NAD(P)^+^/NAD(P)H, nicotinamide adenine dinucleotide (phosphate); NO, nitric oxide; NOX, NADPH oxidase; O_2_^•−^, superoxide anion; Prx1, peroxiredoxin 1; ONOO^−^, peroxynitrite; ROS, reactive oxygen species; SOD, superoxide dismutase; TR, thioredoxin reductase; Trx-S_2_, oxidized thioredoxin; Trx-(SH)_2_, reduced thioredoxin.

As a primary source of ROS, mt-ROS exert an essential role in PAH due to their involvement in apoptosis, inflammation, cell signaling, and mitochondrial DNA (mtDNA) damage. Aberrant antioxidants and mt-ROS production are present in PAH upon the conversion to aerobic glycolysis [121]. In PAs and PASMCs of fawn hooded rat (FHR)-PH, depressed mt-ROS and decreased superoxide dismutase 2 (SOD2, an intramitochondrial antioxidant enzyme) have been correlated with normoxic HIF-1α activation and inhibition of oxygen-sensitive voltage-gated K^+^ channel expression [50]. Conversely, in PAECs from persistent PAH of the newborn (PPHN), mitochondrially localized manganese superoxide dismutase (MnSOD) expression and activity are inhibited, contributing to oxidative stress and endothelial NO synthase (eNOS) dysfunction. MnSOD transduction of PPHN-PAECs decreases mitochondrial superoxide generation and improves eNOS function and the PA relaxation response [122]. Increased mitochondrial superoxide production is observed in the chronic hypoxia-induced PAH model, triggering HIF-1α stabilization, metabolic reprogramming, and increased intracellular calcium concentration, thereby triggering HPV [121,123,124]. Discrepancies within published work may exist due to different experiment conditions, species variation, and the complicated interaction between hypoxic signaling and other triggers of PAH. Another important source of ROS is the activity and expression of monoamine oxidase A (MAO-A), which is increased in pulmonary vasculature and RV of PAH patients and experimental PH models. Pharmacological inhibition of MAO-A improves RV afterload and pulmonary vascular remodeling in SuHx rats by reducing pulmonary vascular proliferation and oxidative stress [125]. In addition, accumulating evidence indicates that mt-ROS, NOXs, and other sources of ROS act in concert to stimulate oxidation, which is further facilitated by reduced antioxidant ability (SOD, catalase, glutathione S-transferase, glutathione peroxidase, and thioredoxin) in PVCs in PH (Figure 2) [126,127].

## 5. Ferroptosis and Lipid Peroxidation in Pulmonary Hypertension

Ferroptosis is a form of regulated cell death induced by the oxidative disturbance of the microenvironment within the cell. This microenvironment is controlled by GPX4 and can be restrained by lipophilic antioxidants and iron chelators. Bioinformatic analysis in lung samples from IPAH patients found the activation of the ferroptosis pathway [128,129]. Administration of deferoxamine, an iron chelator, prevents the development of PH and pulmonary vascular remodeling in rats under hypoxic exposure. In vitro experiments demonstrate that various iron chelators suppress ET-1, platelet-derived growth factor (PDGF), and FBS-induced PASMC proliferation [130]. However, the precise pathological role of ferroptosis in PH development remains unclear.

Lipid peroxidation is evidence for oxidative stress in PH. Samples from PAH patients display increased lipid peroxidation. Specifically, levels of F_2_-isoprostane, a specific lipid peroxidation product found in the urine of patients with PH, are 2.3 times that of healthy control groups. Changes in mPAP and PVR after NO inhalation are correlated with basal F_2_-isoprostane levels [131]. There are also research reports demonstrating that urinary F_2_-isoprostane levels are independently related to the mortality of PAH patients [132]. BMPR2 mutated transgenic mice show a pronounced increase in isoprostanes [133]. Isoprostanes act on the pulmonary vasculature in a variety of ways, including pulmonary vasoconstriction [134], the release of ET-1 [135], and hypertrophy in smooth muscle [136]. Thus, they play an essential mediator role in PH pathologies. Likewise, the level of malonic dialdehyde, an end product of lipid peroxidation in the plasma from IPAH patients, is higher than in healthy volunteers [137]. Together, these studies indicate that PH is causally linked to enhanced lipid peroxidation.

## 6. Mitochondrial Iron and Calcium Homeostasis

**Figure 3:**
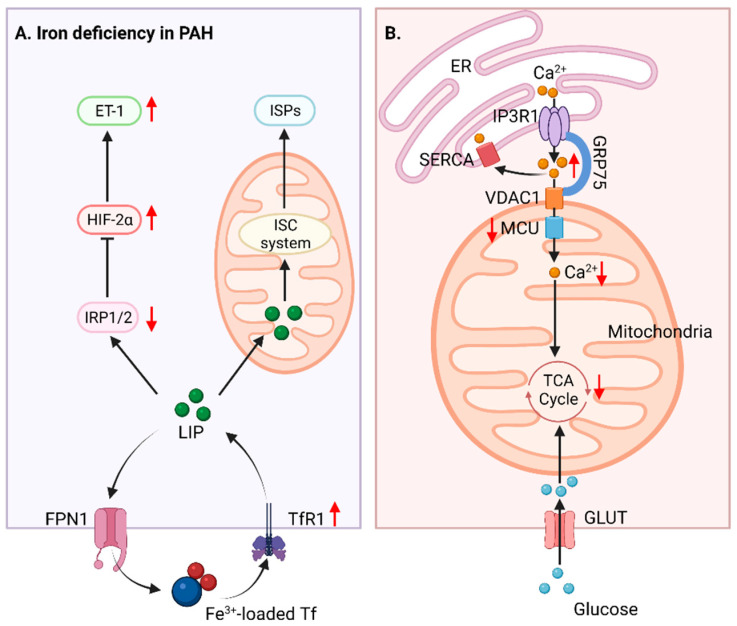
**Mitochondrial iron and calcium imbalance in PH.** (**A**) Iron uptake and distribution are regulated by IRP, FPN1, and TfR1. In hypoxic and iron-deficient contexts, IRP1 is reduced to stabilize HIF-2α in vascular cells and subsequently induces the expression of ET-1, which regulates pulmonary vascular contraction and the proliferation of SMCs, cardiomyocytes, and fibroblasts. (**B**) Ca^2+^ transfer between ER and mitochondria is mediated by a multiprotein complex composed of IP3R1 in ER or RYR in SR, GRP75, and VDAC1 in OMM, and MUC in IMM. Mitochondrial Ca^2+^ uptake affects mitochondrial metabolism and promotes glucose oxidation via stimulating the Krebs cycle. The loss of MCU in PAH decreases the mitochondrial Ca^2+^ and simultaneously increases cytosolic Ca^2+^, inhibiting PDH and promoting a shift to glycolysis. ER: endoplasmic reticulum, ET-1: endothelin-1; FPN1: ferroportin 1, GLUT: glucose transporter, GRP75: 75 kDa glucose-regulated protein, HIF-2α: hypoxia-inducible factor 2α, IMM: inner mitochondrial membrane, IP3R1: inositol 1,4,5-trisphosphate receptor type 1, IRP1/2: iron regulatory protein 1/2, ISC: iron-sulfur cluster; ISPs: ISC-containing proteins, LIP: labile iron pool, MCU: mitochondrial calcium uniporter, OMM: outer mitochondrial membrane, RYR: ryanodine receptor, SERCA: sarco and endoplasmic reticulum calcium transporting ATPase, SR: sarcoplasmic reticulum, TCA: tricarboxylic acid, Tf: transferrin, TfR1: transferrin receptor 1, VDAC1: voltage-dependent anion-selective channel protein 1.

Mitochondrial iron metabolism participates in oxygen transport, DNA synthesis, energy production, and mitochondrial function. Recently, the relationship between PAH pathobiology and iron deficiency has emerged (Figure 3). Several clinical studies have observed that iron deficiency is linked to poorer clinical outcomes in patients with various forms of PAH [138,139,140,141]. Moreover, it has been suggested that iron supplementation may improve HPV and hypoxic PAH [142,143], and clinical trials are underway to evaluate the effect of intravenous infusion of iron in PAH patients [144,145]. Iron deficiency may promote the development of PAH in an HIF-dependent manner as iron is an essential cofactor in maintaining prolyl hydroxylase domain protein (PHD) activity [146]. In mammals, the iron regulatory proteins (IRPs) regulate iron homeostasis and control the expression of iron uptake proteins such as that of transferrin receptor 1 (TfR1), divalent metal transporter 1 (DMT1), the iron storage protein ferritin, and in some cells, the iron export protein ferroportin (FPN) [147,148]. Among these, targeted deletion of IRP1 has been reported to enhance iron deficiency, induce ET-1 expression, and enhance the development of PAH by increasing HIF-2α expression [149,150]. Conversely, TfR1 deletion attenuates pulmonary vascular remodeling and protects against the development of hypoxic PAH. Interference of TfR1 reduces platelet-derived growth factor-BB (PDGF-BB)-stimulated PASMC hyperproliferation [151]. Iron deficiency elevated right ventricular systolic pressure (RVSP), PA muscularization, and RV hypertrophy exist in inducible SMC-specific fpnC326Y gene knock-in mice. Importantly, the replenishment of iron prevents and partially reverses PAH development [152]. Increased understanding of the role of iron in the pathophysiology of PAH may provide new therapeutic targets for reversing pulmonary vascular or RV pathologic remodeling.

Calcium homeostasis plays a pivotal role in metabolic processes including mitochondrial respiration, oxygen-sensing, and apoptosis [153]. Under physiological conditions, the accumulation of Ca^2+^ in mitochondria stimulates oxidative metabolism by activating TCA cycle enzymes [154]. Several studies have demonstrated that a rise in cytosolic Ca^2+^ and decreased mitochondrial Ca^2+^ are directly related to PAH pathogenesis [155,156,157]. Increased cytosolic Ca^2+^ can cause PASMC contraction, proliferation, and migration, leading to pulmonary vasoconstriction and vascular remodeling. Enhanced store-operated Ca^2+^ entry (SOCE) and receptor-operated Ca^2+^ entry (ROCE), and voltage-dependent Ca^2+^ entry, contribute to the elevation of cytosolic Ca^2+^ in PASMCs from IPAH patients [157]. Different regulatory systems control mitochondrial Ca^2+^ in the progression of PAH (Figure 3). For example, in PAs from the MCT-PH rat model and PAH-PASMCs, the mitochondrial calcium uniporter (MCU) complex dysfunction is associated with increased cytosolic Ca^2+^, decreased mitochondrial Ca^2+^, and inhibited glucose oxidation, potentially promoting PAH development. Restoring MCU function reverses mitochondrial Ca^2+^ and the PAH-associated PASMC phenotype [158]. In addition, uncoupling protein 2 (UCP2) has been shown to facilitate Ca^2+^ influx from the endoplasmic reticulum (ER) to mitochondria. PASMCs deficient in UCP2 display reduced levels of mitochondrial Ca^2+^, suppressed glucose oxidation, activated HIF-1α, and pulmonary vascular remodeling [159]. The mitochondrial permeability transition pore (mPTP) also mediates calcium flux across the IMM [160]. mPTP opening exists in PAH [161]. Blockade of mPTP using cyclosporine A (CsA) prevents MCT-induced mitochondrial damage in RV and improves RVH [162], indicating that mitochondrial permeability transition participates in the pathogenesis of PH by affecting mitochondrial calcium homeostasis. Collectively, these reports support an important role for mitochondrial calcium homeostasis in the development of PAH, and may offer new therapeutic modalities.

## 7. Mitochondrial Biogenesis and Mitophagy in Pulmonary Hypertension

The maintenance of functional mitochondria depends on mitochondrial quality control systems including mitochondrial fusion, fission, and biogenesis to sustain mitochondrial function and mitophagy for degradation of damaged mitochondria [163]. Mitochondrial biogenesis requires the participation of nuclear-encoded mitochondrial proteins (NEMPs) delivered to mitochondria. The critical transcription factors including nuclear respiratory factor-1 (NRF1), peroxisome proliferator-activated receptor gamma coactivator 1-α (PGC-1α), and estrogen receptor increase the number of NEMPs [164]. Several PH models have proven that PH is accompanied by a reduction in mitochondrial biogenesis genes and mass. Specifically, SuHx-PH models exhibit reduced gene expression of PGC-1α, peroxisome proliferator-activated receptor α (PPAR-α), and estrogen-related receptor α (ERR-α) in RV tissues [165]. Likewise, PGC-1α mRNA and protein levels were significantly decreased in PASMCs from PAH patients, MCT-PH, and SuHx-PH rats [166]. Conversely, PGC-1α is induced and activates mitochondrial biogenesis, thereby enhancing PASMCs proliferation in cultured rat PASMCs under an early stage of hypoxic exposure [167]. The differences in mitochondrial biogenesis may be related to experimental conditions and the complex interaction between hypoxic stress and other triggers of PAH. Recently, studies have demonstrated that several mediators including NO and estrogen modulate mitochondrial biogenesis in PH. In lamb models of PPHN, inhaled NO treatment partly restored impaired mitochondrial biogenesis and function [168]. In addition, estrogen administration in SuHx-treated ovariectomized female rats showed improved RV function, which was achieved by protecting the mitochondrial function and biogenesis [169].

Mitophagy is an autophagic response targeting mitochondria with apparent characteristics of selective autophagy. Mitophagy is a complex process initiated by changes in the mitochondrial membrane potential, such that PTEN-induced kinase 1 (PINK1) stably aggregates on the OMM, recruits parkin RBR E3 ubiquitin-protein ligase (PRKN), and degrades the damaged mitochondria. According to several studies, mitophagy is involved in the pathological process of lung diseases including PH. Specifically, the expression of PINK1 and PRKN is increased in lung tissues from IPAH patients, mice exposed to chronic hypoxia, and PASMCs under hypoxic exposure [170,171]. *Pink1*^-/-^ mice show reduced pulmonary vascular remodeling and improved hypoxia-induced RV dilatation, while there is no obvious difference in RVSP [171]. In addition, the role of UCP2-mediated mitophagy in intermittent hypoxia-induced PH and right heart dysfunction has been found. Endothelial-specific Ucp2 knockout mice exhibit more severe PH accompanied by increased mitophagy. Knockdown of PINK1 in the endothelial Ucp2 knockout mice alleviates the development of PH [172]. Therefore, future studies on the role of mitophagy in the pathogenesis of PH are required.

## 8. Sphingolipid Metabolism in Pulmonary Hypertension

Sphingolipids are bioactive lipids in cell membranes, which participate in various physiological functions. The metabolism of sphingolipids occurs in subcellular locations, where mitochondria are critical for the activities of ceramide-producing enzymes [173]. According to reports, the sphingosine kinase (SphK)/sphingosine-1-phosphate (S1P)/sphingosine-1-phosphate receptors (S1PR) pathway is involved in a variety of lung diseases including PAH [174]. SphK1 and its product S1P, but not SphK2, expression is higher in the lung tissues and PASMCs from PAH patients as well as in lung and PA tissues from hypoxia-induced PH rodent models. Knockout or pharmacologic inhibition of SphK1 in mice prevents hypoxia-mediated PH development, while SphK2-deficient mice show no difference. The loss of sphingosine-1-phosphate lyase 1 (SGPL1) leads to an increase in S1P levels, which contributes to the development of hypoxia-induced PH. Mechanically, the pathway stimulates PASMC proliferation via the ligation of S1PR2, thereby promoting pulmonary vascular remodeling [175]. Similarly, activation of the SphK1–S1P axis contributes to PDGF and transforming growth factor β1 (TGF-β1)-induced PASMC proliferation [176,177], indicating the role of SphK1–S1P in the pathogenesis of PAH. In addition, selective SphK1 inhibitor PF-543 administration in hypoxia-induced PH mice model leads to reduced RVH. Still, it does not affect pulmonary vascular remodeling, which is due to the compensation of SphK2 for the loss of SphK1. Comparatively, another SphK1 and ceramide synthase (CerS) inhibitor RB-005 fails to improve RVH, implying that it eliminates the beneficial effect of SphK1 inhibition in hypertrophy upon synchronous inhibition of CerS [178]. Tabeling et al. demonstrated the role of sphingolipids in HPV. Under hypoxic exposure, SphK inhibitor SKI II and S1P receptor 2/4 (S1P2/4) antagonist JTE-013 suppresses HPV. In parallel, blockade of SphK or S1P2/4 weakens neutral sphingomyelinase (nSMase)-induced pulmonary vasoconstriction [179], identifying the role of sphingolipids in HPV.

## 9. Therapeutic Potential and Challenges

Given the metabolic reprogramming theory in PAH, several preclinical and early clinical studies have focused on targeting glucose metabolism to treat and improve PAH. An inhibitor of PDK, DCA has been found to ameliorate hemodynamic changes and RV function in PH animal models, increasing mitochondrial respiration, reducing mPAP and PVR, and improving functional capacity in IPAH patients [49,52]. The 3-Bromopyruvate (3-BrPA), a selective HK-2 inhibitor, can inhibit PASMC proliferation and migration by attenuating glycolysis, and is effective in reversing hypoxia-induced pulmonary vascular remodeling in rat models [180]. Targeted inhibition of PFKFB3 with 3PO suppresses glycolysis and completely prevents PAH in rats treated with SuHx [62,181]. Suppression of enolase 1 using AP-III-a4 (ENOblock) inhibits the metabolic shift to glycolysis and prevents hypoxia-induced PAH in mice and SuHx PH rats [182]. Thus, we believe that targeting glucose metabolism is likely to be an effective therapeutic strategy in PAH.

If we focus on the metabolic substrate switch theory in PAH, FAO has been studied in potential targeted therapies. Inhibitors of FAO, Ranolazine, and Trimetazidine improve cardiac index and treadmill walking distance and attenuate exertional lactic acidemia in an RVH rat model [99]. Phase I safety study of Ranolazine (NCT01757808) was conducted in 12 patients with PAH and those receiving background PAH therapies, but consistent therapeutic effects were inconclusive [183]. The safety and efficacy of Ranolazine have been further assessed in a prospective 3 month open-label pilot study involving 11 patients with symptomatic PAH. In addition to being safe, Ranolazine administration improved World Health Organization (WHO) functional class, reduced RV size, improved RV function, and showed a trend toward improved exercise capacity on bicycle echocardiography. However, Ranolazine did not alter invasive hemodynamic parameters [184]. The effect of Ranolazine on RV function was investigated in a multicenter study (NCT01839110). Ranolazine treatment in precapillary patients with PAH receiving stable PAH-specific vasodilator therapies showed improved RV function, left ventricular (LV) end-diastolic volume, and biventricular stroke volumes [185]. The efficacy of Trimetazidine has been studied in a phase II trial (NCT02102672), but no data have yet been published [186]. Another study reported that the glutamine antagonist DON inhibited glutaminolysis, caused a reciprocal increase in glucose oxidation, elevated cardiac output, reduced RVH, and improved RV function in an RVH rat model [91]. A scavenger of reactive lipid peroxidation products, 2-hydroxybenzylamine (2HOBA), showed normalization of glutamine metabolism and prevented BMPR2 mutant-mediated development of PAH in mice [90].

As the disruption of redox homeostasis participates in the pathophysiology of PAH, the use of antioxidants as potential therapeutics in PAH is attractive. Accumulated evidence has shown the beneficial effects through the utilization of agonists of antioxidant systems, inhibitors of ROS production, and modulators of ROS-induced toxicities in the PAH animal models. Treatment with SOD-mimetic metalloporphyrin Mn(III)tetrakis (4-benzoic acid) porphyrin (MnTBAP) reverses the hyperproliferative PAH phenotype, reduces mPAP and RVH, and improves exercise capacity in a PAH rat model [187]. Likewise, another SOD mimetic drug, TEMPOL, enhances vasoconstrictor reactivity and attenuates the development of chronic hypoxia and SuHx-induced PAH. However, it does not improve RVH and pulmonary arterial remodeling [188]. Clorgyline, an inhibitor of MAO-A, ameliorates RV dysfunction and pulmonary vascular remodeling through inhibiting oxidative stress in SuHx-treated rats [125]. An inhibitor of NOX4 GKT137831 reduces hypoxia-induced H_2_O_2_ release, the hyperproliferation of PVCs, and attenuates hypoxia-induced PAH in mice [189]. In patients with PAH, supplementation with coenzyme Q (CoQ), which is required for mitochondrial function and antioxidant reactions, showed a beneficial effect for heart function and red cell production by improving mitochondrial and redox metabolism [190]. To date, many treatment efforts to improve PAH outcomes by restoring the redox balance have been made; however, complex results are emerging due to the possibility of generating reductive stress to disturb redox homeostasis.

These reports of metabolic intervention successes open a new venue for PAH therapies. However, further experimental and clinical studies are needed to determine whether metabolic interventions will reverse the process of pulmonary vascular and RV remodeling and even improve survival. Furthermore, in the context of current PAH therapeutic approaches, combination therapies should be explored, considering the dysfunction of interactional metabolic pathways across multiple tissues. In summary, therapeutic technologies based on mitochondrial metabolism pathways in PAH are promising but need further exploration and development given, disease heterogeneity.

## 10. Conclusions

Accumulating evidence has identified mitochondrial metabolic abnormalities as contributors to the development of PAH. Alterations in cellular metabolic processes in PAH include changes in glycolysis and PPP, increased glutamine utilization and FAO. Generation of secondary signaling messengers such as ROS, iron, and Ca^2+^ is closely associated with abnormal signal transduction underlying PAH development. Additionally, several enzyme systems and key transcription factors such as HIF-1α have been reported to mediate mitochondrial metabolic pathways in PH. Hence, it is necessary to further explore the specific mechanisms underlying disorders in mitochondrial metabolism, the key molecular triggers and regulatory checkpoints of these pathways, and interconnectedness of these aberrant metabolic pathways in disease processes. Thus, new theoretical basis and targets for the diagnosis and treatment of PAH will be provided.
